# Rapid detection of human herpes virus by next-generation sequencing in a patient with encephalitis

**DOI:** 10.1186/s12985-019-1205-x

**Published:** 2019-08-17

**Authors:** Yu Zhang, Kun Hong, Yueli Zou, Hui Bu

**Affiliations:** Department of Neurology, The Second Hospital of Hebei Medical University, Shijiazhuang City, Hebei Province China

**Keywords:** Human herpes virus type 1, Viral encephalitis, Next-generation sequencing

## Abstract

**Background:**

The inflammatory or non-inflammatory changes caused by the virus entering the nervous system and related tissues are central nervous system virus infections. Viral infection is a common infectious disease of the central nervous system, of which herpes simplex virus encephalitis is the most common. However, conventional laboratory techniques to detect an infectious agent are difficult to achieve etiological diagnosis.

**Case presentation:**

Here we present a patient with severe and progressive encephalitis, requiring diagnosis of the specific pathogen to guide clinical treatments.

**Conclusions:**

Application of next-generation sequencing provided a quick and definite diagnosis of the etiology of encephalitis and enabled our patient to be treated appropriately.

## Background

Encephalitis is a complicated clinical syndrome for which current diagnostic testing of infectious, autoimmune, and neoplastic causes often yields no identifiable etiology. More than half of encephalitis cases are unexplained [1]. Encephalitis can be caused by infection with microbial agents, such as bacteria, fungi, viruses and parasites [2, 3]. Viruses are important pathogens, yet are often a poorly understood cause of encephalitis [1]. Among the causes of viral encephalitis, herpes simplex virus type 1 (HSV-1) infection is the most common cause of sporadic encephalitis [4]. When HSV-1 infects the central nervous system, typical symptoms and signs occur including Encephalitis, meningitis, seizures, language impairment, memory disturbance [5]. Notable imaging findings of herpes simplex encephalitis (HSE) are asymmetric abnormalities in mesiotemporal lobes, orbitofrontal lobes, and insular cortex with edema, possible restricted diffusion or haemorrhage [4]. The disability and mortality rate of HSE is high. Prognosis of HSE mainly depends on rapid diagnosis and early initiation of treatment. Timely and rapid diagnosis is hindered by the lack of available assays to survey the full range of common, rare, or unknown agents responsible for encephalitis. Next-generation sequencing (NGS) makes pathogen identification without a priori knowledge possible [6]. Here we introduce NGS to identify an infectious agent in a complicated case of encephalitis with unknown etiology.

## Case presentation

**Fig. 1:**
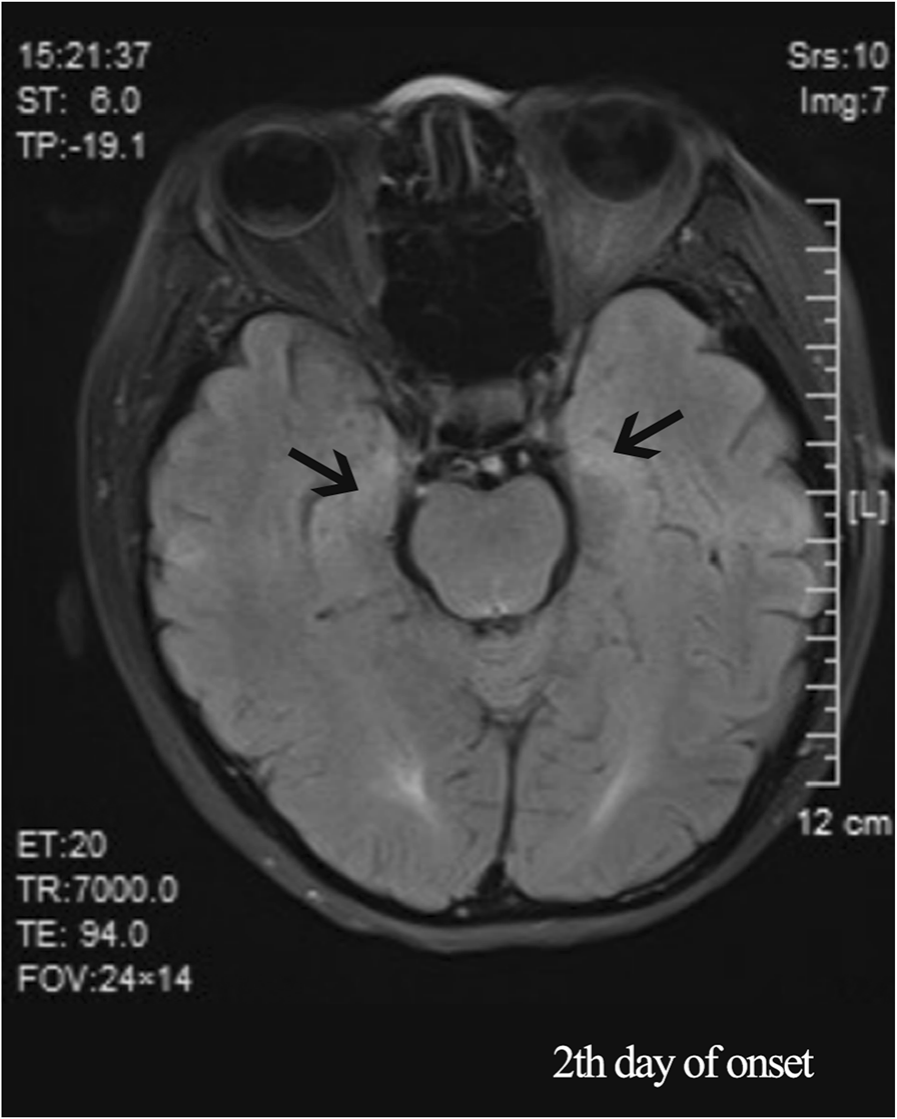
Brain MRI showed fluid attenuated inversion recovery (FLAIR) hyperintense lesions (white arrows) in bilateral cingulate gyrus and bilateral temporal cortex

A 47-year-old woman complaining of bradyphrenia for 3 days was admitted to our hospital on August 29, 2016, after losing consciousness. She suffered from ulcerative colitis for 18 years, receiving treatment with oral hormones (Methylprednisolone, 16 mg daily) and Isoniazid (0.3 g, daily). There was no history of smoking, coronary artery heart disease, diabetes mellitus or hypertension. The patient initially presented with bradyphrenia, shaking of the left lower limb and incontinence for 3 days. The patient suffered a sudden fever the day before admission and then fell unconscious, upon which she was admitted to the Neurology department of a regional hospital. Brain magnetic resonance imaging (MRI) was performed, which showed fluid attenuated inversion recovery (FLAIR) hyperintense lesions in the bilateral cingulate gyrus and bilateral temporal cortex (Fig. 1). Magnetic resonance venography of the head was normal. She was treated with aspirin and atorvastatin calcium for suspected cerebral infarction.

**Fig. 2:**
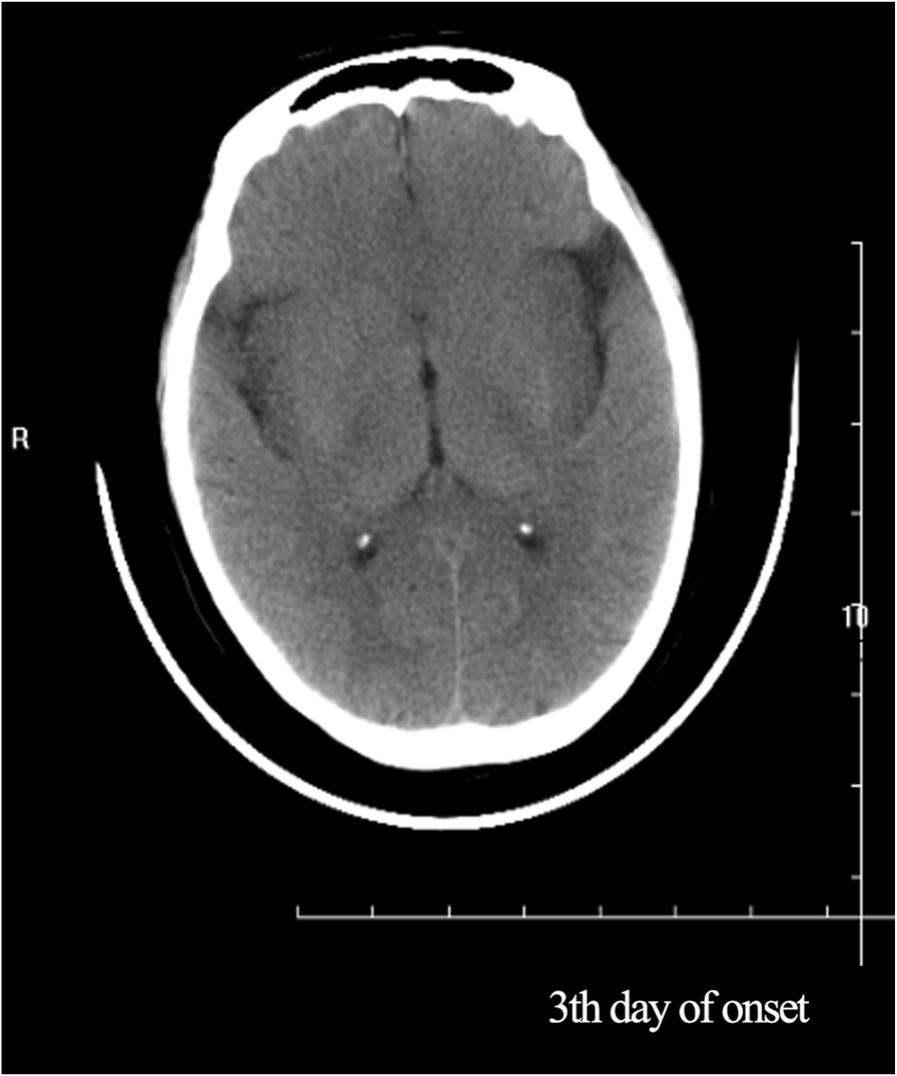
Computed tomography of the brain revealed hypodense lesions in the bilateral insula and bilateral frontal cortex, corresponding with limbic encephalitis

**Fig. 3:**
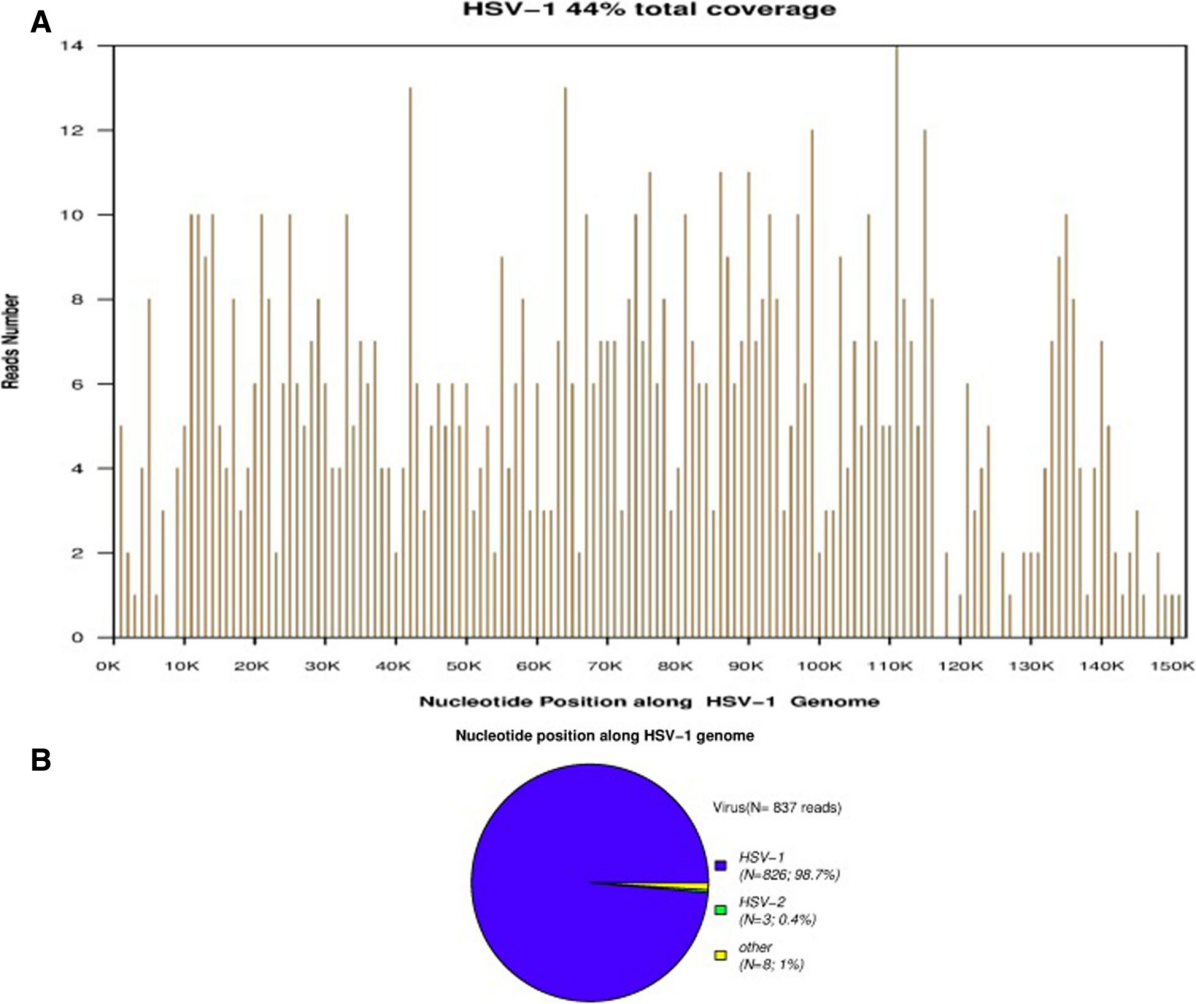
**a**, **b** NGS results of pathogen identification. In our patient, 98.7% of viral reads corresponded to HSV-1, with coverage of 44%

**Fig. 4:**
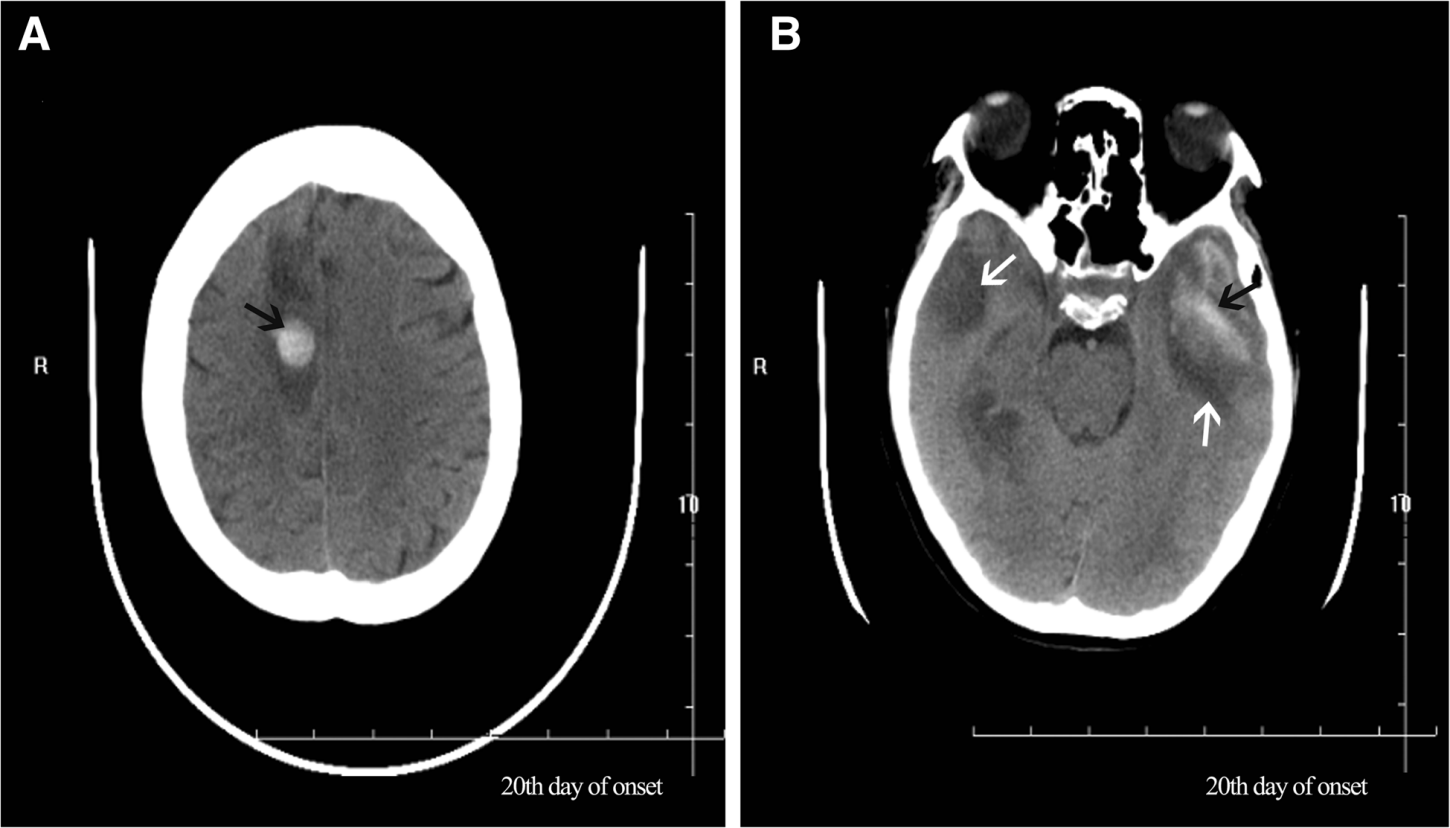
**a** CT scan of the brain showed multiple hypodense lesions (white arrows) in the right frontal lobe, right insula, bilateral temporal lobe and left basal ganglia, including right frontal lesion hemorrhage (black arrow). **b** CT scan of the brain showed the range of hypodense lesions in bilateral temporal lobes (white arrows) had increased, and hemorrhage within the left lower temporal lobe hypodense lesion (black arrow)

**Fig. 5:**
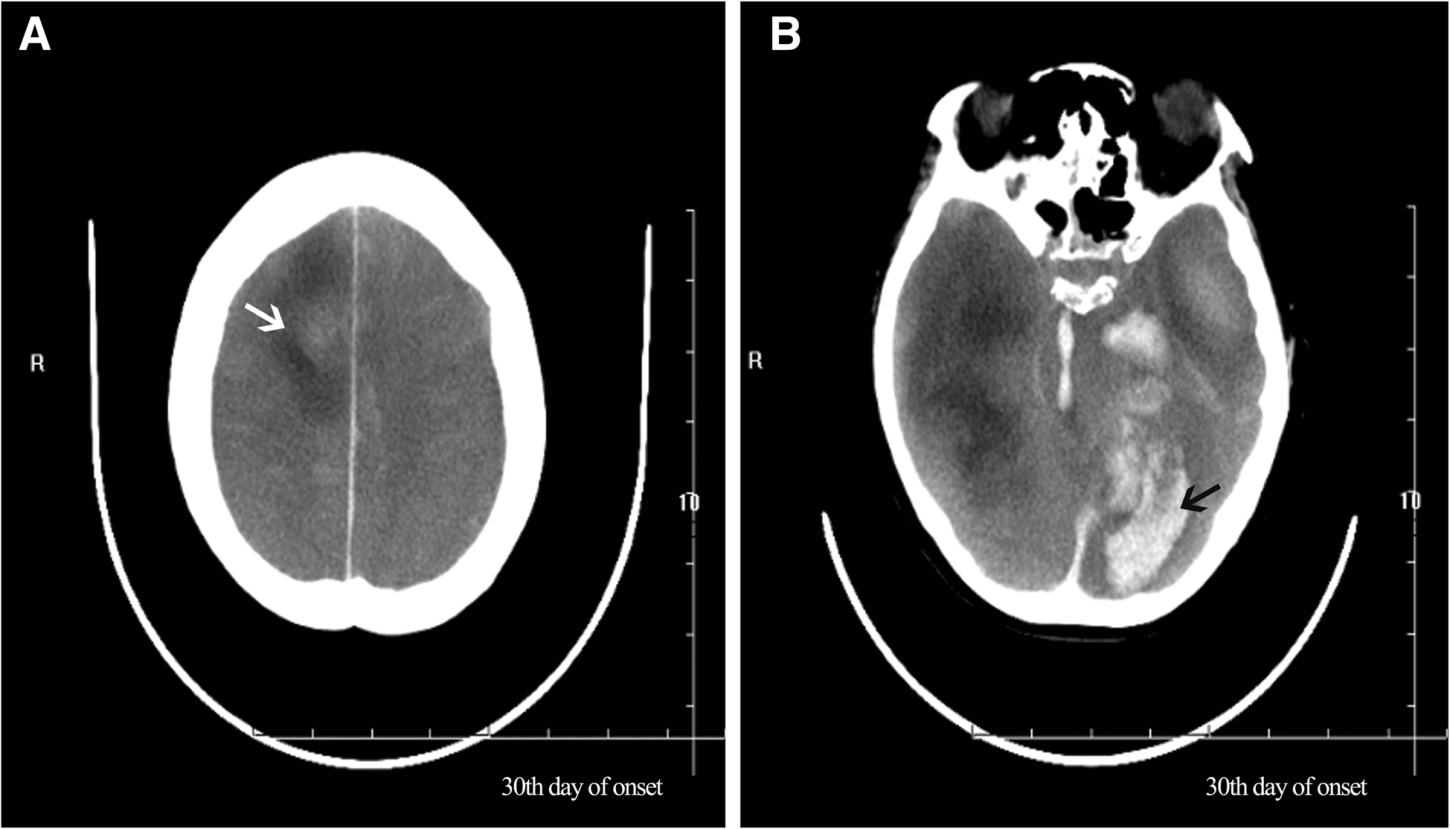
**a**, **b** A repeated CT scan of the brain showed the following: 1. The hemorrhages and densities (white arrow) in the right frontal lobe and left temporal lobe were decreased; 2. A new massive brain hemorrhage (black arrow) can be seen in the left occipitoparietal and left basal ganglia, and hematoma broken into side ventricles; 3. The midline deviated from the falx cerebri to the right side; 4. Subarachnoid hemorrhage was apparent

As the patient’s condition did not improve, she was transferred to our hospital. Physical examination on admission revealed comatose state (Glasgow Coma Scale [GCS] 3), stiff neck, and no voluntary movements. In terms of laboratory findings on admission, routine blood, general hematological and biochemical tests showed no abnormalities, and inflammatory markers, such as erythrocyte sedimentation rate, were normal. A lumbar puncture was performed. The cerebral spinal fluid (CSF) opening pressure was 85 mmH_2_O. Routine and biochemical testing of CSF identified the following: proteins 0.55 g/l (0.20–0.40); leukocytes 1 × 10^6^/l (0.0–15.0); glucose 3.2 mmol/l (2.50–4.50); chlorine 131.9 mmol/l (120–132). CSF cytology showed abnormal cytology, with the presence of 35 lymphocytes and a monocyte. Epileptiform abnormal discharge and diffuse slow wave was observed by electroencephalogram. The patient’s condition deteriorated rapidly. As her oxygen saturation continued to decline, she was placed on a mechanical ventilator via intratracheal intubation. Since the patient’s condition failed to improve, she was transferred to the neurological intensive care unit. Meningoencephalitis was suspected, so she was treated with foscarnet sodium (3 g, daily), methylprednisolone pulse therapy (500 mg, daily for 5 days), intravenous immunoglobulin (20 g, daily for 5 days) and other supportive treatments. However, the patient’s condition remained refractory to treatment. Computed tomography (CT) of the brain revealed hypodense lesions in the bilateral insula and bilateral frontal cortex, corresponding with limbic encephalitis (Fig. 2). Laboratory examination revealed autoimmune encephalitis and paraneoplastic syndrome-related tests in both serum and CSF to be normal. Polymerase chain reaction (PCR) assay for herpes simplex virus type 1 and herpes simplex virus type 2 DNA came back negative in CSF. Considering the poor effect of antiviral treatment, next-generation sequencing (NGS) of CSF was used for the detection of pathogens. In total, 5.5 million reads were obtained by NGS, of which 837 were identified as viral, with a detection time of 48 h. HSV-1 DNA was identified in the CSF. The number of identified unique reads mapped on the HSV-1 genome sequence was 826, making up 98.7% of the viral reads. The coverage of the identified HSV-1 genome was 44%, with depth values of 1. The number and percentage of unique reads, coverage, and depth of the identified HSV-1 DNA sequences are presented in Fig. 3a, b. Upon diagnosis with HSV-1 encephalitis, the patient was started on intravenous acyclovir (500 mg, three times daily) and foscarnet sodium (3 g, three times daily). A repeated CT scan of the brain showed multiple hypodense lesions in the right frontal lobe, right insula, bilateral temporal lobe, and left basal ganglia, including right frontal lesion hemorrhage. On the 14th day after admission (Fig. 4a), in view of her deteriorating condition, she was again treated with intravenous immunoglobulin (20 g daily for 5 days). A repeated CT scan of the brain, performed approximately 20 days later, showed the range of the hypodense lesion in the bilateral temporal lobes was increased, and hemorrhage within the left lower temporal lobe hypodense lesion, compared with the previous CT scan (Fig. 4b). After active treatments, the patient’s condition was not markedly improved. On the 28th day after admission, a repeated CT scan of the brain showed the following: 1, the hemorrhages and densities in right frontal lobe and left temporal lobe were decreased; 2, a new massive brain hemorrhage in the left occipitoparietal and left basal ganglia, and hematoma broken into side ventricles were observed; 3, the midline deviated from the falx cerebri to the right side; 4, subarachnoid hemorrhage (Fig. 5a, b). Family members abandoned treatment on day 46 after admission, and the patient died after discharge.

## Methods

### Sample collection and information

The patient with clinically suspected viral encephalitis admitted to second hospital of Hebei medical university in August 2016 was included in this study. Metagenomic sequencing was performed using previously published methods [7] on CSF from a clinically-indicated lumbar puncture, performed on hospital day 5. CSF was collected in accordance with standard procedures, snap frozen and stored at − 20 °C. Tis study was approved by the Institutional Review Board of PUMCH and Beijing Genomics Institute, Shenzhen (IRB No. JS-890). The use of patients’ clinical data and CSF sample for this study was approved by Te Ethics Committee of PUMCH. Written informed consents were obtained from all patients or their legal surrogates.

### DNA extraction, library construction, and sequencing

DNA was extracted directly from the clinical samples using the TIANamp Micro DNA Kit (DP316, Tiangen Biotech, Beijing, China). DNA libraries were constructed through and repaired adapter added overnight, and by applying polymerase chain reaction amplification to the extracted DNA. Quality control was carried out using a bioanalyzer (Agilent 2100, Agilent Technologies, Santa Clara, CA, USA) combined with PCR to measure the adapters before sequencing. DNA sequencing was then performed using the BGISEQ-100 platform (BGI-Tianjin, Tianjin, China).

### Data treatment and analysis

High-quality sequencing data were generated after filtering out low-quality, low-complexity, and shorter reads. To eliminate the effect of the human sequences, the data mapped to the human reference genome (hg19) were excluded using a powerful alignment tool called Burrows-Wheeler Alignment. The remaining data were then aligned to the Microbial Genome Database, which includes bacteria, viruses, fungi, and protozoa. Finally, the mapped data were processed by removing duplicate reads for advanced data analysis.

A nonredundant database that included all the published genomes of microorganisms was downloaded from the National Center for Biotechnology Information (fp://fp.ncbi.nlm.nih. Gov/genomes/). The database used for this study contained 1,492 bacteria, 2,686 viruses, and 60 species of fungi that can cause infections in humans, and 33 species of protozoa related to human diseases. The depth and coverage of each species were calculated using SoapCoverage from the SOAP website (https://github.com/sunhappy2019-8/soap.coverage).

## Discussion

We present here a 47-year-old woman with an 18-year history of ulcerative colitis, receiving treatment with oral hormones, who was recently diagnosed with suspected viral encephalitis. She presented with bradyphrenia and unconsciousness. Nervous system physical examination on admission revealed comatose state (GCS 3), stiff neck and no voluntary movements. Her symptoms appeared abruptly and progressed quickly. She also developed respiratory failure for a short time. MRI of the brain showed fluid attenuated inversion recovery (FLAIR) hyperintense lesions in the bilateral cingulate gyrus and bilateral temporal cortex. A repeated CT scan of the brain showed multiple cerebral hemorrhages. CSF tests showed mild lymphocytosis. We suspected HSV-1 encephalitis, however virus antibody testing did not indicate herpes virus infection. Regardless of anti-cytomegalovirus antibody positivity, we did not consider cytomegalovirus infection. It may be that HSV antigens are similar to those of cytomegalovirus, as HSV-1 was detected in the CSF by NGS. Despite anti-viral treatment and intravenous immunoglobulin, her condition continued to decline until she finally died. We conclude that she suffered from severe encephalitis, and even upon timely and effective treatment; mortality remains high in such cases. Resistance to acyclovir may exist, so the infection cannot be easily controlled.

The etiological agent in this case was not diagnosed by traditional detection methods. Currently, clinical manifestations, imaging studies, and CSF analysis are the basis of the diagnostic approach in viral encephalitis [8–10]. However, it is difficult to identify the virus using traditional detection methods, such as PCR. Therefore, we used NGS to identify the pathogen and initiate appropriate treatment. Novel DNA sequencing techniques, referred to as next-generation sequencing (NGS), provide high speed and throughput which can produce an enormous volume of sequences [9]. Following the publication of the first article on the use of NGS technology to achieve the pathogenic diagnosis of Leptospira encephalitis, a new era of mNGS technology for the diagnosis of central nervous system infection was initiated [11]. Diagnostic virology is one of the most successful applications for NGS and exciting results have been achieved in the discovery and characterization of new viruses, detection of unexpected viral pathogens in clinical specimen [10]. In early 2019, Professor Michael Wilson’s team has evaluated and confirmed the usefulness of the Metagenomic NGS technology [11]. Nearly half of acute meningoencephalitis cases are not etiologically diagnosed. Owing to the lack of timely and identified pathogen diagnosis, patients are not effectively treated. Even after routine pathogen examinations, nearly one-third of patients with nervous system infections are misdiagnosed. As NGS becomes an increasingly accessible technology, we foresee it is likely to become an essential tool in clinical diagnosis.

## Data Availability

We declared that materials described in the manuscript, including all relevant raw data, will be freely available to any scientist wishing to use them for non-commercial purposes, without breaching participant confidentiality.

## Abbreviations

CSF: Cerebral spinal fluid
CT: Computed tomography
FLAIR: Fluid attenuated inversion recovery
GCS: Glasgow coma scale
HSE: Herpes simplex encephalitis
HSV-1: Human herpes virus type 1
MRI: Magnetic resonance imaging
NGS: Next-generation sequencing
PCR: Polymerase chain reaction
